# “You Can Sit in the Middle or Be One of the Outliers”: Older Male Athletes and the Complexities of Social Comparison

**DOI:** 10.3389/fpsyg.2019.02617

**Published:** 2019-12-03

**Authors:** Sean Horton, Rylee A. Dionigi, Michael Gard, Joseph Baker, Patti Weir, Jordan Deneau

**Affiliations:** ^1^Department of Kinesiology, Faculty of Human Kinetics, University of Windsor, Windsor, ON, Canada; ^2^School of Exercise Science, Sport and Health, Charles Sturt University, Port Macquarie, NSW, Australia; ^3^School of Human Movement and Nutrition Sciences, University of Queensland, Brisbane, QLD, Australia; ^4^School of Kinesiology and Health Science, York University, Toronto, ON, Canada

**Keywords:** Masters athletes, competition, role models, aging, qualitative research, sport promotion

## Abstract

Sporting events for older adults are proliferating in both popularity and participation numbers, mirroring the growth that is occurring globally with an aging population. Preliminary evidence indicates that older athletes have a tendency to compare themselves (in terms of their performance, participation, and aging) to inactive older adults deemed “worse-off.” Our aim was to examine the stories and experiences of older, male Masters athletes, not only in terms of their own lives and in relation to others but also in the broader context of current (neoliberal) policies that promote sport across the lifespan. We use social comparison theory to interpret our findings and highlight the strengths and limitations of social comparison as a psychological strategy. For this study, 17 male competitive athletes (age range from 70 to 90 years) who participated in either the 2013 or 2017 World Masters Games were interviewed as part of a larger project on the meaning of sport in their lives. Seven different sports were represented, and participants hailed from multiple countries. Within the interpretive paradigm, we used qualitative methods to interview each participant, analyze individual transcripts, and develop common themes across the data set to address the aforementioned aims. Our two major themes were, *Sport as social comparison:“It’s the competitive nature”* and *Downward comparisons*. A number of participants commented on the nature of sport, and competitive sport in particular, as being important to their motivation to train and prepare. Within the theme of *Downward comparisons*, we established two categories: *Resisting loss* and *Assigning blame*. While downward comparisons were used by our participants to separate themselves from other seniors of the same age, thereby bolstering their sense of self, participants also tied those comparisons to neoliberal notions of individual and moral responsibility for health. Participants believed that compromised health was due to individual negligence and bad decisions, with little reference to uncontrollable factors, such as non-modifiable risk factors for disease, disability, and/or socioeconomic status, which could be affecting people’s lives or decisions. Ultimately, our findings show that the useful psychological strategy of social comparison for maintaining a positive sense of self and performance may also have some negative individual and societal consequences.

## Introduction

“It put me upon reflecting how little repining there would be among mankind at any condition of life, if people would rather compare their condition with those that were worse, in order to be thankful, than be always comparing them with those which are better, to assist their murmurings and complaining”—Daniel Defoe, Robinson Crusoe (Defoe, 1719).

Sporting events for older adults are proliferating in both popularity and participation numbers (Weir et al., 2010; Jenkin et al., 2017), mirroring the growth that is occurring globally with an aging population. Masters sporting events are an increasingly common form of later-life leisure, although older adults are still vastly underrepresented compared to other age groups in both physical activity levels (Troiano et al., 2008; Statistics Canada, 2015; Gomes et al., 2017) and sport participation statistics (Canadian Heritage, 2013; van Uffelen et al., 2015; Eime et al., 2016).

Masters athletes are individuals who take part in age-defined events in which competition is normally delimited by 5- or 10-year age groupings. The age at which one becomes eligible is sport specific; for most sports, one can participate at the Masters level after the age of 25 or 30, and it is not uncommon for athletes to compete into their 80s, 90s, and even over 100 years of age (Pfister, 2012). Masters sport has experienced explosive growth in recent years (Weir et al., 2010; Gard et al., 2017). The World Masters Games (WMG), for example, is a quadrennial event that started in 1985 in Toronto, Canada with approximately 5,000 participants (Weir et al., 2010). It has grown to the point that it is the largest multisport event in the world, typically hosting 20,000 to 30,000 athletes (International Masters Games Association, 2016). To put this in context, the 2016 Summer Olympic Games in Rio, Brazil had approximately 11,000 athletes competing (International Olympic Committee, 2018).

Masters sporting events, and their participants, are challenging long-standing notions about what is possible in later life in terms of human performance and leisure interests. It is often assumed in the exercise sciences, media, and policy that sport participation in later life is a positive example of modeling human potential across the lifespan, which should be celebrated and promoted to all, especially in developed countries (Coakley, 2015; Gard et al., 2018). However, our preliminary work (e.g., Gard et al., 2017; Horton et al., 2018) has revealed a moralizing component to Masters sport, such that it appears to cue the denigration of less physically active older adults who are often “blamed” for burdening the health care system. This way of thinking aligns with economic efficiency and individual responsibility for health, which underpins what has been termed a neoliberal shift in sport and health policies and promotion (see Gard et al., 2017 for a discussion of such research)^1^. This shift sees sport being used as a tool in social policy to help solve population health issues (see Gard and Dionigi, 2016). To critically examine assumptions surrounding sport in later life and to build on previous findings, our aim was to understand the stories and experiences of older Masters athletes (of their performance, participation, and aging), not only in terms of their own lives and in relation to others but also in the broader context of current (neoliberal) policies that promote sport across the lifespan^2^. We used social comparison theory to interpret our findings and highlight the strengths and limitations of social comparison as a psychological strategy.

## Literature Review and Theoretical Framework

Currently, there is a small, albeit growing, body of literature examining Masters athletes from biomedical and psychosocial perspectives. However, qualitative research specific to the psychosocial experience of sport participation for older adults is limited relative to that in the biomedical and quantitative domains. From a biomedical perspective, Masters athletes have been regarded as exemplars of aging success or models of human potential (Hawkins et al., 2003; Cooper et al., 2007; Louis et al., 2012). For example, older strength and endurance-trained athletes often measure superior to their age-matched, less-active peers on physiological characteristics such as muscle mass (Wroblewski et al., 2011) and maximal oxygen uptake (Trappe et al., 2013). However, the lens of biomedical aging success (i.e., Rowe and Kahn, 1987, 1997) has been frequently critiqued for taking a narrow focus on what constitutes happiness and satisfaction in later life, especially considering its relative neglect of subjective aging experiences (Glass, 2003; Martin et al., 2015; Martinson and Berridge, 2015). In response, Geard et al. (2017) proposed a more multidimensional model with which to view aging success, incorporating physical, psychological, cognitive, and social functioning criteria. Based on their review, the authors suggested that Masters athletes may represent this more broad definition of successful aging.

In addition to the physical benefits of competitive sport, Masters athletes appear to receive unique positive psychological outcomes, such as motivation for goal-oriented, health-enhancing behaviors (Baker et al., 2010), positive cognitive outcomes such as mental flexibility (Pesce and Audiffren, 2011), and positive social outcomes such as a sense of community (Lyons and Dionigi, 2007). In addition, Dionigi et al. (2013) showed how sport participation can be used as a strategy to simultaneously resist, redefine, and accept the aging process. Although research examining the non-physical outcomes derived by Masters athletes is preliminary, it does suggest that those who compete in events like the Masters games derive multidimensional outcomes above and beyond what would be accrued from what might be considered more “traditional” exercise (Dionigi et al., 2011a,b).

A recent systematic review has advanced knowledge related to the psychosocial outcomes of older adults’ sport participation (Gayman et al., 2017). Broadly, existing literature suggests competitive sport bestows older adults with aging-related, cognitive/perceptual, emotional, social, and motivational benefits (Gayman et al., 2017). However, due to a paucity of data, the authors were unable to conclude whether these benefits were solely related to participation in sport. What does seem to be clear is that the manifestation of psychosocial outcomes through Masters sport is complex and varied. Of particular importance to the current paper is the preliminary observation that older male athletes are likely to use participation in sport as a means of distinguishing and distancing themselves from perceived “usual agers” on the basis of physical abilities (Eman, 2011, 2012). This tendency demonstrates the broader inclination of older adults to compare themselves to those they deem “worse-off” for self-enhancement purposes (Lockwood et al., 2005). We were interested in this particular pathway that older male athletes tend to use to derive positive psychosocial outcomes from Masters sport and the resulting implications for the individual and society.

### Social Comparison Theory, Aging, and Sport

The proclivity of individuals to compare themselves to others is well established (e.g., Festinger, 1954; Wills, 1981; Wood, 1989; Lockwood et al., 2005; Gerber et al., 2018). The social comparison process involves individuals evaluating their opinions and abilities with one another for self-evaluation, self-improvement, and self-enhancement purposes (Wood, 1989; Gerber et al., 2018). These motives may be influenced by the social environment and are multidirectional, such that individuals may compare “upward,” “downward,” or “laterally” (Wood, 1989; Gerber et al., 2018). Upward comparisons tend to manifest as an evaluation with a person or group perceived as superior or “better-off” (Wheeler, 1966; Collins, 2000; Gerber et al., 2018), whereas downward comparisons tend to manifest as an evaluation with a person or group perceived as inferior or “worse-off” (Wills, 1981; Gerber et al., 2018). Lateral comparisons are made with others who are perceived as relatively equal in status; however, the three-choice paradigm (i.e., upward, downward, and lateral comparison) is understudied relative to the two-choice paradigm (i.e., upward and downward comparison; Gerber et al., 2018). The degree to which one uses either an upward or downward comparison strategy may be explained by regulatory focus theory, which suggests individuals possess a promotion or prevention orientation (Higgins, 1997, 1998). Those with a promotion orientation tend to focus on pursuing positive and desirable outcomes, whereas those with a prevention orientation tend to focus on avoiding negative and undesirable outcomes. Furthermore, some individuals are simply more inclined to make social comparisons than others (Gibbons and Buunk, 1999; Buunk and Gibbons, 2007; Merriman et al., 2019). The degree to which one demonstrates a predilection to socially compare is termed social comparison orientation (SCO; Gibbons and Buunk, 1999).

Findings from a recent meta-analysis (Gerber et al., 2018) revealed that individuals are generally more likely to make upward rather than downward comparisons, and the response is typically a contrast and subsequent decrease in self-evaluation. Regardless of the direction of comparison, those who are being compared to may be conceptualized as “role models.” Role modeling is rooted in social cognitive theory (Bandura, 1977), which emphasizes “vicarious experiences” as influential to one’s attitudes, beliefs, and behaviors. Changing attitudes and beliefs about aging and sport may be an effective means through which to increase physical activity participation in later life (Sarkisian et al., 2005). Role models, whether positive or negative, have the potential to stimulate positive behavior change (e.g., adopting a “healthier” lifestyle) by altering others’ attitudes and beliefs and motivating individuals to set and achieve goals (Morgenroth et al., 2015). In the context of one’s health, individuals with a promotion focus have a tendency to compare upward and are often motivated to achieve relevant health characteristics of “positive” role models (e.g., a friend who exercises regularly and consumes a balanced diet; Lockwood et al., 2005). On the other hand, individuals with a prevention focus have a tendency to compare downward and are often motivated to avoid health characteristics of “negative” role models (e.g., a sedentary colleague with multiple chronic health conditions; Lockwood et al., 2005). Importantly, the mechanisms, variables, and outcomes of role modeling manifest differently based on setting (e.g., educational, occupational, and achievement settings) and role aspirant profile (e.g., gender, race, and age; Morgenroth et al., 2015). These nuances are critical given that role model research with a focus on the unique considerations among older adults is sparse, with the majority of work focusing on younger cohorts (e.g., Zirkel, 2002; Chlosta et al., 2012; Chen et al., 2013). Additionally, less is known about role models in the context of encouraging sport participation in later life, providing part of the backdrop for our current article.

There has been speculation that social comparison regulatory orientation will change over the course of one’s life. Specific to health and aging, Lockwood et al. (2005) demonstrated that as people age, they exhibit a greater tendency to compare downward to those in perceived poor health states. This may be due to the fact that the priority of older adults gradually shifts from optimizing, to maintaining, and ultimately to preventing the erosion of skills, abilities, and health (Lockwood et al., 2005). During youth, individuals tend to compare upwards, looking to role models in anticipation of improvements and advancements (Heckhausen, 1997; Ogilvie et al., 2001), and it is widely acknowledged that young people benefit from having positive role models (Zirkel, 2002; Chlosta et al., 2012; Chen et al., 2013). For example, Chen et al. (2013) demonstrated that having supportive role models who help reframe stressors and promote optimism was associated with reduced cardiovascular risk among low socio-economic status youth. For seniors, however, loss prevention may be just as important as promotion (Lockwood et al., 2005); thus, the emphasis turns to preserving what they have, as opposed to focusing on further gains. For older adults, negotiating later life becomes more of a promotion-prevention balance, and they may benefit from both positive and negative role models (Lockwood et al., 2005; Horton et al., 2008, 2013).

Downward comparisons in later life may have real psychological utility. Wills’ (1981) basic principle of downward comparison states that, “Persons can increase their subjective well-being through comparison with a less fortunate other” (p. 245). In essence, by focusing on and comparing themselves to an individual less fortunate, one may feel more reassured and gratified with their own abilities (Wills, 1981; Frieswijk et al., 2004). This process can be fueled by negative affect, low self-esteem, ego threat, and/or declining status (Wills, 1981; Gerber et al., 2018). Downward comparison principles appear to be particularly relevant with respect to older adults who, one could argue, are a “threatened” population (Horton et al., 2007; Dionigi, 2015). That is, seniors will inevitably experience age-related changes across many dimensions in their lives, including health status. When these dimensions are particularly self-relevant, individuals are inclined to see themselves as superior to, and distinguished from, others by means of downward comparison (Wood, 1989).

Many Masters athletes regard sport and physical competence as integral to their identity, with those at the extreme preferring to die rather than be sedentary (Dionigi et al., 2013; Dionigi, 2017). This might help explain why Masters athletes tend to make comparisons to, and at times disparage, other seniors who they perceived as lazy, sedentary, and morally inferior (Gard et al., 2017). This disparaging is perhaps also a reflection of the internalization of negative aging stereotypes in Western society, such as later life as a time of poor health, loneliness, and dependency (Ory et al., 2003; Horton et al., 2007). The processes and outcomes of downward social comparison seem to be riddled with complexities, such that these strategies may result in both benefits and problematic consequences for individuals and society.

The targets of downward comparisons may experience threats to their well-being. Wills (1981) noted that, “People consistently select safe targets—groups or persons whom the dominant culture considers acceptable to derogate” (p. 246). In the context of the current sport and active aging promotion climate, the “dominant culture” may be understood as Masters athletes, whereas the “targets” may be understood as inactive older adults. Gard et al. (2017) argued that recent neoliberal “sport for all” policy trends, which use sport as a solution to population health issues, have led to a normalization of participation in high-level, strenuous, competitive physical activity in later life. In other words, these widespread beliefs about the benefits of sport participation for one’s health are constructing societal expectations for older adults to regularly participate in sport and structured exercise for the health of the nation, which may lead to “othering” seniors who do not partake (Dionigi, 2017; Gard et al., 2017, 2018). Such policies potentially conflate the *efficacy* of sport and physical activity for improving one’s health (of which there is considerable evidence) with the *effectiveness* of sport and physical activity promotion in increasing participation levels and improving “population health” (of which there is no clear evidence; see Weed, 2016).

If we ignore the personal, social, and circumstantial determinants of healthy behaviors, we fail to realize that many seniors lack the means, access, ability, and interest to participate in sport, exercise or regular physical activity (Phoenix and Grant, 2009; Dionigi, 2017). As a result of their non-participation, sedentary individuals are often viewed by active older adults, and in the media and policy, as a burden on society in general, and health care systems more specifically. If current sport policy and promotion trends continue, Dionigi (2017) believes these inactive “pariahs” may be further victimized, stigmatized, and medicalized, reinforcing feelings of guilt and self-blame in regard to health outcomes. Moreover, in a heightened cultural and political context of sports’ utility across the lifespan, it is important to consider how Masters athletes, who often place such great importance on high levels of physical performance, will adjust psychologically when they inevitably succumb to age-related physiological limitations (Dionigi, 2016, 2017). Ultimately, it is important to deepen understandings of Masters sport participation in an attempt to maximize the positive and minimize the negative multidimensional psychosocial outcomes.

Recent evidence highlighted older female athletes’ (aged over 70 years) derived multifaceted benefits from competitive sport, as well as their tendency to subtly denigrate those who do not participate (Horton et al., 2018). However, research specific to older male athletes in this regard is sparse. Gayman et al.’s (2017) systematic review examining the unique psychosocial outcomes of sport involvement in later life outlined five studies exclusive to older male athletes. Two were case studies; qualitative interviews with an 88-year-old American runner (Roper et al., 2003) and a 68-year-old American tennis player (Langley and Knight, 1999). The remaining studies examined primarily psychological outcomes of older males’ participation in competitive tennis, including locus of control and achievement motivation (Rotella and Bunker, 1978); collision avoidance skill, perception, and knowledge of action capabilities (Lobjois et al., 2008); and coincidence-timing, performance, visuomotor delay, and ability to adapt to changes in stimulus velocities (Lobjois et al., 2006). Four studies in Gayman et al.’s (2017), review included both men and women, one of which (Eman, 2012) highlighted the tendency of older male athletes to make comparisons with those that they perceived as less physically capable. Given this observation, and observations made in our previous work (i.e., Gard et al., 2017; Horton et al., 2018), we considered it important to explore this particular aspect of the male experience. The extent, frequency, and somewhat unsympathetic nature of the older men’s social comparisons were noteworthy. Social comparison theory permitted us to scrutinize the male experience in this particular athletic and socioeconomic context more thoroughly, as well as highlight the negative implications of downward social comparisons by contextualizing the study within the concept of neoliberal health policies. Notably, the men in Eman’s (2012) study were from Sweden and primarily involved in track and field, swimming, and skiing. Advancing knowledge and informing stakeholders of the psychosocial aspects of Masters sport from the perspective of a broad range of participants who vary in age, nationality, and chosen sport is timely.

### Study Aims

This article focuses on interviews with older male participants in the WMG, which is part of a larger study on the meaning of sport in the lives of older adults (see Gard et al., 2017; Horton et al., 2018). Our focus for this article was in examining the data specific to how athletes spoke about their own sport participation, not only in terms of their own lives and in relation to others but also in the broader context of current (neoliberal) policies that promote sport across the lifespan. To that end, we investigated the thoughts, feelings, and attitudes of a particular group of older adults who were not the focus of our publications cited above – male Masters athletes aged 70–90 years – toward their own physical performance, participation, and aging. We use social comparison theory to interpret our findings and highlight the strengths and limitations of social comparison as a psychological strategy. The findings are intended to contribute to a more in-depth understanding and informed discussion of the psychological ramifications and the societal implications specific to how older adults view performance, active aging, and sport participation, thereby contributing to the literature across sport psychology, health promotion, and public policy.

## Methodology

### Research Paradigm

This study was broadly situated within the interpretive paradigm and used qualitative methods to make sense of the multiple and subjective meanings that people brought to their experiences and social world (Denzin and Lincoln, 2008). Specifically, social comparison theory was used as our guiding framework for interpreting the findings in this qualitative study. We drew broadly on the seminal works of several foundational theorists in the field (Festinger, 1954; Wheeler, 1966; Wills, 1981; Wood, 1989), were informed by Gerber et al.’s (2018) meta-analysis on social comparison research, and guided by Lockwood et al.’s (2005) work on age and health-related regulatory orientations within the social comparison paradigm (as detailed above). Lockwood et al. (2005) is particularly relevant given their emphasis on health and aging, allowing us to examine the complexities of social comparison among Masters athletes in the wider health promotion context.

Qualitative research, with its focus on human subjectivity, is sensitive to the complex experience of aging, sport, and society (Dionigi, 2006, 2016; Phoenix and Grant, 2009; Phoenix, 2018). To our knowledge, no study has qualitatively examined the stories of older male athletes specifically framed within social comparison theory and with consideration of the broader health promotion context. Therefore, we employed semi-structured interviews and purposive sampling to collect data, followed by an analysis and interpretation of data to address our aims.

### Reflexivity Statement

As authors, we recognize that participants attach multiple, dynamic, and often contradictory meanings to their own aging. By listening to our participants’ stories, we attempted to understand how active older men make sense of their lives, actions, and attitudes toward their sporting experiences. Participant responses were part of their personal, historical, and cultural context, and notably, so were our interpretations of them. All authors are long-term residents of Western nations (i.e., Canada and Australia), as were our participants, yet we are primarily middle-aged. We are all trained in the broad field of exercise and sport sciences, with more specific training in psychology and sociology relevant to the aims and scope of this study. Moreover, we each brought subtleties in our perspectives and approaches to the team, which allowed us to critically question each other throughout the research process (Smith and McGannon, 2018). This is particularly relevant given that we, as authors, were interpreting the words of our participants. As part of the qualitative approach and data analysis process, we have chosen certain passages to represent their thoughts and feelings, and we fully acknowledge our bias in that selection process. We realize that readers may interpret passages differently than the authors; thus, we have provided a number of verbatim quotations to not only justify our interpretations but also give the reader an opportunity to make their own meanings of the words participants have employed.

### Participants

Participants for this particular component of our project were older, male competitive athletes who participated in either the 2013 (Turin, Italy) or 2017 (Auckland, New Zealand) WMG. Our previous work has used subsets of data from 2013 WMG participants (e.g., Gard et al., 2017 focused on men and women aged 60 years and over, while Horton et al., 2018 focused on women only). The present study adds to our ongoing project by presenting previously unreported data from the 2013 WMG, coinciding with the addition of data from the 2017 WMG and an explicit focus on social comparison theory and its implications among older male athletes.

Each participant was interviewed once, as explained below. We analyzed the interview transcripts of 17 male athletes ranging in age from 70 to 90 years. These participants were selected through purposive criterion-based sampling (Patton, 2002) based on specific criteria, including: age (70+ years of age), gender (male), language spoken (English), and competing in the WMG. Seven different sports were represented, which included badminton, basketball, lawn bowls, swimming, tennis, track and field, and weightlifting. Sixteen of the men identified as Caucasian, while one participant identified as Asian. Participants hailed from six countries: Australia, Canada, England, Germany, New Zealand, and the United States. As a group they were highly educated, although the range reported was considerable, from just a few years of formal education (i.e., elementary school) to a PhD. The majority were fully retired, with one continuing to work full-time and one part-time. Professions listed included civil servant, engineer, farmer, management, mechanic, physician, pilot, professor, teacher, and sales. Eleven indicated that they were married and four were widowers, whereas two did not indicate their marital status. Ethics approval was obtained from a University’s Research Ethics Board and all participants provided informed consent. In order to protect participant confidentiality, all were given pseudonyms for the purposes of data presentation.

### Procedure/Interview Format

The first five authors of this article participated in the development of the interview protocol and conducted interviews at the respective 2013 and 2017 WMG events. We approached athletes at their competition venues to ask if they would be interested and willing to partake in an interview. The interviews themselves were conducted in a quiet space away from the sporting action, and we organized these interviews around participants’ competitive schedules. A semi-structured interview format was utilized (Patton, 2002) which gives the interviewer the flexibility to probe participant responses for further detail. The topics included are sporting background/sport-life trajectory; training/preparation; motivation; competition; barriers to participation; role models; and thoughts on non-participation in sport and sports promotion. Examples of the questions we asked included: What role does sport play in your life? What is it about “competition” that attracts you? What inspires you (are you inspired by anyone? Do you have any role models?)? Why do you think many older people do not participate in sport? Interviews typically lasted from 30 to 60 min, although some lasted upward of 90 min. The ensuing dialogue was digitally recorded and subsequently transcribed.

### Data Analysis

All authors were involved in the data analysis process. Specifically, the second and third author led the analysis of the 2013 data, whereas the first and last author led the process for the 2017 data. Together, the data were initially analyzed inductively to determine themes, which were then deductively interpreted using social comparison theory. That is, we used an inductive approach for the coding and analysis of the transcripts, where content-driven thoughts or ideas were identified as “meaning units” (Côté et al., 1993, 1995). Meaning units from different interviews (i.e., intratextually; Maykut and Morehouse, 1994) deemed sufficiently similar were identified and constantly compared against one another until main and secondary themes were established, and data become saturated to the point that nothing new was materializing in the analysis. Next, the themes were interpreted using the guiding framework of social comparison theory. Finally, the findings were discussed in the broader context of psychosocial and societal implications of sport and health promotion in later life, particularly as they pertain to neoliberal health policies.

### Trustworthiness

Within the interpretive paradigm used for this study, several strategies were employed to ensure data trustworthiness, including triangulation to specifically enhance credibility. In particular, *analyst triangulation* was demonstrated by using multiple experienced qualitative researchers to interpret findings (Patton, 2002). While having more than one interviewer/analyst necessarily resulted in some variability in how the interviews were conducted and interpreted, every effort was made, through frequent quality control meetings and discussions among the research team, to ensure all of the key concepts were covered with each participant, and that consensus was reached on the themes. At the same time, interpretive research acknowledges that bias is inevitable because the “biographically situated researcher” is involved in every stage of the interpretive process and this reality should be embraced (Denzin and Lincoln, 2003, p. 30). During our meetings, we discussed the interview process, shared our experiences, questioned one another, self-reflected, and made slight adjustments when necessary. Moreover, purposive sampling was employed to address potential issues of transferability (Patton, 2002). We have attempted to clearly define our participants so that our verbatim quotes may be interpreted specific to older male athletes in relation to the context in which the data collection occurred and not necessarily generalized beyond the sample.

## Findings

Two major themes emerged in the analysis: *Sport as social comparison:* *“It’s the competitive nature”* and *Downward comparisons*. Within the theme of *Downward comparisons*, we established two categories: *Resisting loss* and *Assigning blame*.

### Sport as Social Comparison: “It’s the Competitive Nature”

A number of participants commented on the nature of sport, and competitive sport in particular, as being important to their motivation to train and prepare. This went beyond what they perceived more traditional exercise could provide, and participants talked about working harder than they might otherwise, with competition serving as the key motivator. Neil (74, weightlifting) described the benefits he derives specific to competitive sport: “Really, the competition gives me more of an incentive to work out, you have an incentive to keep your body in shape but I don’t think I would do it to the level that I do without competition.” We also asked participants why they think Masters sport is growing in popularity. In response, Wayne (76, tennis) compared the adrenaline rush he gets from sport to a drug.

I think people are realizing that the potential to be bedridden is there. I think the heart attacks are also there, and so on. I think they’re starting to realize…and every heath professional will tell you that active sport is the way to go. So, I also take up dancing, and that’s very enjoyable. It’s very social as well. But sports give you the really head-on adrenaline that you can’t get from other drugs.

Nathan (75, swimming) distinguished between “serious” swimmers who compete and recreational or “fitness” swimmers, drawing a sharp line between the two in terms of their approach in the pool. Here, we begin to see how a competitive context allows for and promotes social comparison.

For me, the competing really makes me work harder, and it makes you do it right. I see a lot of fitness swimmers, and they make illegal turns. They make “short turns” occasionally, which you just don’t do if you’re serious.

Nathan clearly felt that competitive swimming not only makes him work harder but also enforces discipline that recreational swimmers forego. He went on to say that there is something very different about people who participate in competitive sport and that it is fundamental to their make-up.

Some people just don’t ever seem to want to compete. They are just not made to compete. For me, it’s something I want and like to do. I suspect you must hear something like that from most of the people that are serious competitors. It has to be…A lot of people don’t want to do that. I have tried to convince people that I swim with who don’t compete that they should try competing. I get almost NO takers, it just doesn’t happen…They just don’t have this internal drive.

In this way, Nathan discriminates between competitive athletes and those who, for whatever reason, do not want to partake in competitive sport. Consequently, there appears to be a form of denigration at work, as he laments their lack of “internal drive,” without any recognition of factors outside of their control, such as (dis)ability, socioeconomic or health status, or cultural background, which could be affecting their lack of participation.

Other participants talked specifically about winning. That aspect of competitive sport, establishing winners and losers, and keeping score, was an important component to them. Ian (70, badminton) explained that “it’s the competitive nature, when you play sport the competitive nature is what motivates you, and you are always wanting to win.” Davis (70, basketball) noted that “I like to compete, so I’m always ready to count score. I have to count score.” In a more general sense, we see this from Barry (87, swimming), who compares his athletic prowess to others, and the positive effect that has on him. “I get a thrill, I get an urge of increased thoughts about myself, thinking that I can do this where others perhaps cannot and I relish in the effort of staying young.” It appears that Barry’s “increased thoughts” bolster his sense of self through direct comparison to others, which was a common feature of participant responses, providing the focus of the second theme. Essentially, it is important to emphasize that Masters sport seems to provide a fertile ground for social comparisons, given it is fundamentally a competitive context. To the men in this study, participation is not enough, rather winning and pushing for a personal best are expected and respected, which can exclude those who do not share these competitive ideals. Therefore, this theme has shown comparison *within* the Masters sport context, whereas the next theme focuses on how athletes extended these tendencies by comparing themselves with the stereotypical “inactive” older person.

### Downward Comparisons

This major theme was divided into two subthemes: *Resisting loss* and *Assigning blame*. Both of these subthemes consist of our participants juxtaposing their abilities and capacities with others of a similar age. Comparing themselves with others who were worse off was the dominant tendency and appeared to bolster their own sense of self. Inherent within this, downward comparison strategy was a subtle but unmistakable disparaging of those who do not engage in physical activity.

#### Resisting Loss

A striking aspect of this subtheme was not that participants extolled the benefits of sport and exercise, but the extent to which they paired that notion with how much better they are faring than other people their age. By remaining so physically active, they perceived that they are avoiding the quality of life declines that they associate with the majority of their peers. Participants often made explicit comparisons between their own health and activity levels with what most other people their own age were experiencing. This is expressed in a somewhat benign fashion by Neil (74, weightlifting), who noted: “The physical rewards are very great, I see so many people my own age that have difficulty getting around and it’s very rewarding to feel better.” Somewhat more critical was Lee (72, badminton), who distinguished between his approach and what others his age were doing.

I don’t want to go to an RV park and a bunch of old people are sitting around in a hot tub complaining about, “oh my aches and pains”…most of my time I spend with young people so at my club where I open up the gym and play there is nobody else my age so I compete with the young people.

There are two interesting meanings in this statement – that there is no one at Lee’s gym his age to compete against, so he seeks out younger competitors and the stereotyping of low-income older adults with various ailments. Lee goes on to say:

I think the physical and the fitness is the most important but I was just sitting there with my mixed doubles partner and I said “this is really nice to be here with a bunch of people who participate in sports and have an active mindset.” I would hate to go to some old folks’ retirement community and sit around and do nothing. I couldn’t stand that.

Masters Games provides Lee with access to people who have a similar “mindset” with respect to sports and physical activity. Once again, he makes the comparison to what he sees as perhaps more typical of people his age – sitting around and doing nothing. That is, internal attributes, such as lack of motivation or drive, are being judgmentally assigned to those who do not participate in sport, which aligns with current sport promotion messages and related policies that assume everyone can play sport (e.g., “sport for all” philosophies that underlie the International Masters Games Association; www.imga.ch).

Similarly, Mark (72, badminton) contrasts his life-long devotion to fitness with others who have not done the same, and the consequences he perceives to be associated with those choices.

My aim was, all my life was to keep myself as fit as possible. I’ve seen too many, especially farmers give up farming at 60 or 65 and sit down and most of them only last a few years, they don’t do anything and in less than ten years most of them have died off. I don’t feel like dying just yet.

Mark compared his longevity directly with others he has known who retired, lived a sedentary lifestyle, and subsequently died. Marks’s perception is that his active lifestyle has been the difference between life and death. This presupposes a considerable degree of control over one’s aging process, and also insinuates that people are responsible for their own fate. Essentially, this assumption of choice, self-responsibility for health, and assigning blame for a premature death are key features in neoliberal health policies, which leads us to our second subtheme.

#### Assigning Blame

There are some distinct similarities between Mark’s quote immediately above and what Nathan (75, swimming) states below. Nathan, however, is slightly more explicit in assigning blame and responsibility to those who are inactive and suffering health consequences as a result.

I look at colleagues, who spent their whole life writing papers and doing research…They’re either dead or half dead. They’re struggling to even exist. They’re the same age as me. I attribute [my good health] to the swimming, and the high level of physical activity.

Nathan draws a direct line between behavior and the consequences for health. His choices have led to one outcome, while his colleagues are suffering the consequences for their choices. The implication is that if they have not already died, their quality of life is severely compromised, and the reasons for that are simple and uncomplicated, much like the messages in current sport promotion campaigns that assume we can all “get off the couch” if we so choose (e.g., in 2018 Sport Australia launched “Move it AUS,” which claimed that all Australians, regardless of backgrounds, ages, and abilities, should become physically active and move more often; https://www.sportaus.gov.au/media_centre/news/sport_australia_encourages_all_australians_to_move_it_or_lose_it).

This notion of individual responsibility gets extended, at least for some participants like Barry (87, swimming), to how people can expect to be treated by others in positions of power and authority. Barry asserted that “I think it’s much easier for an administrator to shelve an old person who requires a lot more attention than it is to stand by the side of a currently physical athlete.” Notably, the question that elicited this response from Barry was fairly general in nature, asking him about sport, older people, and participation rates. Thus, we considered this response to be somewhat spontaneous and noteworthy because he believed that he would be favored due to his status as an athlete. The fact that he is an athlete made him more worthy of care, at least in his own mind, than someone more sedentary, who is easier to “shelve,” cast aside, or ignore. This sense of moral superiority appears to be shared by Sam (72, track and field): “There is a spectrum, in everything we do we sit on a bell curve; you can sit in the middle or be one of the outliers. I think for a lot of people the other options are easier.” Sam considers himself to be one of those outliers, and that this is primarily a matter of will power and discipline. To Sam, being an outlier is a conscious choice, rather than the product of circumstances or luck. Through exerting his individual agency, Sam positions himself (rather than *being positioned*, crucially) on that bell curve. Yet again, participation is attributed to internal characteristics and individual choice, with little recognition of social privilege, and being an athlete is positioned as a moral obligation to age well.

Justin (90, swimming) was very explicit in emphasizing both individual responsibility and choice in determining one’s health, and in assigning blame if and when things go wrong.

I mean how could you, when you’re given this beautiful thing called a body in which we live, how could you not look after it and exercise it? This body is the most complicated piece of biological machinery in existence, and it should be cared for. If you don’t, then you don’t deserve to have health. It doesn’t take much to keep it healthy.

According to Justin, compromised health is primarily a choice; in the absence of exercise, you are going to get sick, and you deserve your fate. While downward comparisons are used by our participants to separate themselves from other seniors of the same age, thereby bolstering their self-esteem, they also tie those comparisons to individual responsibility for health, and that compromised health is due to negligence and bad decisions. Poor health is often characterized as a failure of character, and thus deserved, which aligns with neoliberal health policies and marginalizes those who do not have the means, abilities, or desire to make such choices.

We will conclude our findings with a quote from Wayne (76, tennis) who drew a very direct line between sports and health for seniors.

They should be encouraged to play sports. If they are playing sports they are a healthier person. So the hospital bill is not great right? And you see the people in rest homes? Most of them have not played sports. They are sitting there in a dull room, they sit there all day. What fun is that?

## Discussion

It is evident that older male Masters athletes derive a range of unique opportunities and outcomes from their participation in competitive sport. The men in this study displayed a strong desire to train, compete, win, and optimize their performance and health. These traits appear to be common among Masters athletes across a number of studies (e.g., Dionigi et al., 2011a,b; Eman, 2012; Horton et al., 2018), and as a result, older sport competitors are often held up as “exemplars” of human performance and aging success (Geard et al., 2017). In this regard, Masters athletes are challenging common myths and stereotypes that position later life as a time of disengagement, decline, and frailty. As the stories of the men in our study supported, competitive sport also clearly presents a chance for travel, social interaction, and a sense of empowerment (Dionigi et al., 2013). Thus, our participants perceived that Masters sport is distinctively rewarding above and beyond traditional exercise. Common perceptions in Western society tend to align with this belief in the inherent value of sport (Coakley, 2015; Gard et al., 2017). Additionally, it appears that public policy and health promotion efforts have bolstered and encouraged a “sport for all” agenda based, in part, on these assumptions. For those who have the resources and desire to compete, there are undoubtedly good reasons for promoting sport. However, through a critical analysis of our participants’ stories, within a framework of social comparison theory, we aimed to highlight the often-overlooked complexities inherent in Masters sport. In addition, we attempted to highlight the negative implications of downward social comparisons by contextualizing the study within the concept of neoliberal health policies.

### Masters Sport as Fertile Ground for Social Comparison

Previous research has indicated that the highly social nature of sport and sport events largely contributes to its perceived utility (Dionigi et al., 2013; Gayman et al., 2017). This sentiment was certainly expressed by the participants in our study, like Lee, who enjoyed the company of teammates with a similar “active mindset.” Moreover, the competitive aspect inherent in sport may be a particularly potent motivating force among older male athletes. Eman’s (2012) interviews revealed that older male athletes overtly valued results and rankings. Similarly, the men in our study placed great importance on winning and keeping score as central to their drive to stay active through sport. Davis, for example, indicated he must keep score when playing basketball. We believe that this often-beneficial nature of competitive sport may also foster a powerful social comparison environment.

Gerber et al. (2018) reported that social comparisons are strongest when targets are local, personal feedback is received, and when comparers are primed to feel dissimilar. Viewed through a social comparison lens, Masters sport appears to possess these characteristics. Target immediacy describes the tendency for outcomes of comparison (e.g., self-evaluation) to be most pronounced when the comparison is made to others who are spatially and temporally proximal (i.e., local)^3^. Masters sport is an intimate environment where athletes compete against and work with one another closely. Moreover, these relationships are enacted in a context where results and feedback on performance are inherent and ubiquitous. Thus, the locality of others and the competitive environment are logically optimal for frequent and robust social comparison opportunities. The athletes in our study persistently emphasized the social and results-focused aspects of Masters sport, which often came through and were embedded in evaluations with other athletes, peers, and the general population.

When held up as exemplars of active aging (e.g., in the media, policy, and academic literature), Masters athletes are being primed with dissimilarity relative to stereotypically inactive older adults. Feeling dissimilar heightens contrasts of self-evaluation away from the target of comparison (Gerber et al., 2018). It was clear that many of our participants perceived themselves to be different than sedentary others which resulted in self-enhancing contrasts. Ultimately, we deduced that the proximity of targets in the feedback-oriented Masters sport context cued social comparisons *within* Masters sport, and this tendency segued into comparisons *beyond* Masters sport (i.e., the “general” older adult population) when primed with feelings of dissimilarity. However, further research is needed to support and/or refute these postulations.

### Psychological Utility of Downward Comparisons

We have presented preliminary evidence and inferences that sport participation provides older adults with an opportunity to compare themselves to others in somewhat similar circumstances. This applies to a competition itself as measured by wins and losses but often to their age cohort more broadly beyond the sporting arena. Findings have indicated that it is important for some older athletes to see themselves as “better” and “healthier” than their peers, whether this is expressed in an implicit or explicit manner (Dionigi, 2017; Horton et al., 2018). Responses from our participants suggested they rely heavily on “negative role models” to bolster their own psychological mindset and sense of self. Specifically, the older men conceived of comparison targets who were sedentary and in poor health, such as Nathan’s colleagues in academia and Lee’s caricature of a low-income individual with an inactive lifestyle. Those perceived as “worse-off” appeared to provide motivation for our participants to avoid the ill-health and quality of life declines often associated with old age. Thus, responses indicated that a “prevention” mindset with respect to their health was prevalent, perhaps more so than a “promotion” mindset, supporting previous findings and theory among older adults (Lockwood et al., 2005; Horton et al., 2013). Specifically, participant objectives related to stemming losses and minimizing aging-related health decline. While some participants had certain individuals who inspired them, or “positive role models,” downward comparisons to negative role models were mentioned more frequently.

These findings and considerations underline the importance of age differences in social comparison and role model research. In general, individuals tend to make upward comparisons (in contrast to downward comparisons) in social comparison situations (Gerber et al., 2018). However, a lifespan approach to social comparison theory indicates potential nuance. From the perspectives of older male athletes, it appears negative role models provide a non-trivial source of motivation to initiate, sustain, and improve human performance and potential in later years. Although our findings were elicited from the stories of a narrowly defined range of individuals, we believe that there are implications warranting exploration in other cohorts and salient to discussions on sport policy.

The men in this study often acknowledged the “perils” of aging (e.g., declining health) and how they were motivated by sedentary others to mitigate these perils through sport involvement. Lockwood et al. (2005) noted that, “downward comparisons may be a more effective means through which older individuals can adjust psychologically to the aging process.” (p. 386). Depending on the role model profile, upward comparisons may actually threaten individuals, reminding comparers of their “inferiority” (Ory et al., 2003; Lockwood et al., 2005; Horton et al., 2013). “Superstar” and “super-fit” role models perceived as possessing unattainable characteristics and success have been reported to discourage the adoption of healthy behaviors, rather than encouraging them (Lockwood and Kunda, 1997; Ory et al., 2003; Horton et al., 2008). Downward comparisons, on the other hand, may fortify the comparer’s psychological mindset, serving as a reminder of their “superiority” and contributing to feelings of gratefulness (Wills, 1981; Wood, 1989). This, in turn, may attenuate any potential feelings of inadequacy, failure, and resentment of those more fortunate than themselves. In the context of the current push for everyone to be active as they age, downward comparisons may be effective at motivating positive behavior change and reinforcing healthy behaviors (Lockwood et al., 2005). Essentially, downward comparison targets serve as an illustration of the costs of poor lifestyle choices, providing the comparer with a roadmap of what not to do and encouraging healthy habits – a process implicitly expressed by our participants. Further, downward comparisons may also provide utility in the realm of anxiety and stress reduction (Wills, 1981) to a cohort (i.e., older adults) that shares feelings of threat and uncertainty about their current and future status (Horton et al., 2007; Dionigi, 2015).

While this research is still in its early stages, our findings support the observation that older adults have a tendency to make downward comparisons. Advocates might argue in favor of promulgating this strategy as an effective means to cope with aging and promote, maintain, and retain performances near the limits of personal potential. As Daniel Defoe indicated in our opening quotation, a sense of gratitude is enhanced by comparing one’s condition to others who are worse-off. Notably, however, downward comparisons often contain subtle disparagement of others, and while this has been touted as a useful psychological strategy (e.g., Wills, 1981; Frieswijk et al., 2004; Lockwood et al., 2005), there may be problematic individual and societal implications (as detailed below) of comparing downwards.

### Societal Implications of Downward Comparisons

Our participants often attributed the behaviors of their sedentary peers to internal characteristics rather than external factors. For example, Nathan generalized that older adults who do not compete in sport are lacking an “internal drive.” There was a substantial lack of attribution made to sociocultural and environmental determinants of health and aging, which are well established in the literature (e.g., Kerr et al., 2012). The older men felt better about themselves by “othering” inactive members of their age cohort, potentially using an attribution error as their vehicle to do so. Inactive older adults were associated with the “threat” of old age, which allowed the Masters athletes to distance themselves from that threat. From a societal perspective, and in the context of neoliberalism (see Harvey, 2005; Peck, 2010), these psychological tendencies may lead to negative consequences including stigmatization, stereotyping, and blaming of older adults who are unable to, or simply choose not to, engage in traditional forms of physical activity. This potentially sets up an in-group (active) out-group (inactive) bias, in which those who are sedentary are denigrated for their lifestyle (Gard et al., 2017). Many older adults, due to personal, historical, and circumstantial complexities, will not enjoy sport and exercise nor will they desire to partake in these endeavors (Dionigi, 2017). Nonetheless, our participants, along with “sport for all” public policy messages, have insinuated that inactivity in later life is unacceptable (Dionigi, 2017).

More broadly, the stories of our participants align with current neoliberal policies that focus on individual responsibility for health (Gard et al., 2017). As a result, the individual is blamed when they experience poor health, regardless of sociocultural determinants that may have contributed to their circumstances. Dionigi (2017) lists potential concerns with an overemphasis on individual responsibility for healthy aging, including a heightened fear of age-related health declines, an expanding social gap between active and sedentary older adults, and potential reductions in welfare support for those in later life. As sport participation behavior normalizes, these neoliberal attitudes have been reinforced, and in a sense, have become state-sanctioned and part of official policy (Gard et al., 2017). In this context, our participants expressed a degree of moral authority that they believed their active lifestyles granted them. Justin, for instance, stated sedentary individuals do not deserve to be healthy. Notably, the backgrounds of our participants suggest several advantages in terms of ability and opportunity to take responsibility for a physically active and engaged lifestyle. That is, the older male athletes were typically educated, Caucasian, married, and possessed the resources to regularly participate in a costly and time-intensive endeavor (i.e., Masters sport). This draws attention to researchers’ indications that dominant health promotion messages in Western society are most relevant to those who have the backgrounds that our participants possessed (Nettleton, 2006; Gard et al., 2017). Thus, it is feasible that our participants’ profiles contributed to their views being aligned with broader neoliberal health promotion discourses, which emphasize personal responsibility for health and aging, position keeping active as a moral obligation, blame those who are not active for the nation’s rising health costs, and undervalue sociocultural forces that potentially influence “right” lifestyle choices. Therefore, we argue that while negative role models may serve as an effective means to preserve a positive sense of self for Masters athletes, there are distinct implications for those who are the targets of these downward comparisons.

The majority of men in our study placed high value on a “forever young” mentality, such as Barry who stated, “I relish in the effort of staying young.” Declarations such as these plausibly lead to a heightened fear of aging and a fear of ultimately becoming part of this stigmatized group. That is, constant exposure, reference, and internalization of ideals of youthfulness contribute to the social construction and individual conceptualizations of aging as a process to be avoided (Muise and Desmarais, 2010). It is common for groups in Western culture, particularly those with individualistic values (as opposed to collectivist values), to subscribe to notions of biomedical anti-aging (Haboush et al., 2012), and in doing so, they may be narrowing the possibility and probability of aging well. The reality is that many of those who reach an advanced age will experience impaired health (e.g., 82% of Canadians 71 years of age and older reported at least one high-impact, high prevalence chronic health condition; Statistics Canada, 2016). Unless athletes die on the court or in the field of play – a wish expressed by some of our participants and Masters athletes in previous works (e.g., Dionigi et al., 2013) – ill health in old age is almost inevitable. Dionigi (2017) noted that heightened expectations for older adults to remain highly active in later life could result in a senior citizenry that has difficulty coping with an inability to participate when disease and disability inevitably strikes. This concern may be particularly true for older athletes, as they tend to be uniquely invested in their sport endeavors and perceive their athlete status as a key element of their identity (Gayman et al., 2017; Horton et al., 2018). Psychological adjustment may be a challenge for those who are forced to cease sport participation for any number of reasons, including injury, chronic health conditions, and/or financial limitations.

Common to the human condition is a propensity to build our sense of self by comparing to worse-off others (Wills, 1981; Wood, 1989). This is apparent in interpersonal everyday life and on a broader societal scale. The paradox of downward comparisons is that we often need groups to denigrate in order to feel good about ourselves. Instances of illogical in-group/out-group biases are widely available and draw attention to the noted denigration by Masters athletes (and society) toward inactive older adults. For example, our findings support Peck’s (2010) claim that, in the context of neoliberalism, new class divisions can emerge (e.g., the Creative Class), or in our case, the class of active older adults, which can widen the gap between the “haves” and the “have nots” in society. Peck (2010, p. 212) argued that the problem with the Creative Class is that those in this class form “like-minded enclaves, with little concern for the wider social consequences” and often claim that anyone can rise to their level, ignoring barriers caused by social inequality (much like the active older adults did in our study). In terms of health and aging, the problem with such class divisions is that the inactive group (or “underclass”) will be further marginalized and ignored in a neoliberal context which removes public funding and shifts the responsibility of aged care from the state to the individual (Gard et al., 2017). Further research on such emerging examples of social stratification within sport contexts and the associated implications is needed.

Particularly salient to the present time is the pervasiveness of social comparison through social media networks (Lee, 2014). Social media users are given ample opportunities to compare their circumstances with those of others through mediums that often lack important context. Those with a high tendency to compare themselves to others have been shown to experience negative psychological outcomes (e.g., poorer self-perceptions, lower self-esteem) after engaging in social comparisons on social media sites (Vogel et al., 2015). We theorize that Masters sport, like social media networks, provides a context that encourages social comparison. However, a key distinction is that older athletes appear to favor downward comparisons while social media users are disproportionately cued to compare upward (Lee, 2014; Vogel et al., 2015). Since upward comparisons have been found to result in deflating self-evaluations (Gerber et al., 2018), one may see the merit in comparing to those who are “worse-off.” Importantly, we argue that downward comparisons are problematic too, not only to the comparer, but the target of the comparison. Sport has the potential to be a positive experience for older adults, however, its promotion may be problematic when it is viewed as a superior means of later-life leisure, a public health intervention, or as a panacea to combat the “burdens” of an aging population (see Dionigi and Gard, 2018).

## Conclusions

This study elicited the perspectives of older male athletes on their participation in competitive sport. By focusing on the stories and experiences of sport participation among men 70 years of age and older, who represented multiple countries and played a number of different sports, the findings built and expanded upon previous work (e.g., Eman, 2012; Gard et al., 2017; Horton et al., 2018). In particular, our participants simultaneously resisted and reinforced aging stereotypes. These older male athletes indicated the uniquely competitive nature of sport, and their associated participation, as a key factor in achieving high levels of performance in later life as well as combatting pervasive notions of loss, decline, and disengagement in old age. Comparison target immediacy, personal feedback, and dissimilarity priming are fundamental to Masters sport. Downward comparisons represented the predominant comparison approach used by our participants to separate themselves from other, less active seniors of a similar age, thereby bolstering their sense of self. This may represent an effective strategy for older adults to adjust to their own aging and feel grateful for both their enviable circumstances and their abilities. However, a useful psychological strategy for maintaining a positive sense of self may have some problematic consequences at a broader societal level. These comparisons often contained denigration toward inactive older adults and were tied to ideas about individual responsibility for health and the economization of health, such that participants felt compromised health was due to negligence and bad decisions. The “in-group” of active older adults perceived the “out-group” of inactive older adults as stereotypical agers who are burdening the health care system. Our participants tended to overlook a number of complex factors that may have contributed to their fortunate circumstances, a way of thinking that could lead to less societal support and personal empathy for the most vulnerable in society. Ultimately, we recognize the inevitability of social comparison and the various potential merits and drawbacks of both upward and downward comparison strategies. By shedding light on the complexities of social comparison in a group of male Masters athletes, we hope to stimulate further discussions across the fields of sport psychology, health promotion, and public policy in this context.

There are limitations inherent in this study. Notably, participants constituted a predominately Caucasian, highly educated, upper-middle class sample. Although this sample is consistent with the archetypal demographic profile of Masters athletes (Gard et al., 2017), future studies should solicit the views from a more diverse range of participants. It would be beneficial to compare the similarities and differences between typical and minority groups in this cohort. Moreover, garnering the opinions of non-athlete older adults on Masters sport will be important to more fully inform health promotion initiatives. This is especially true considering less active older adults are generally the targets of sport and aging programs (Gard et al., 2017). Most importantly, we encourage the continued gathering and mobilization of older adults’ stories so that their voices may contribute to shaping directions for health policies, sport, and aging agendas, and modeling human performance across the lifespan.

## Data Availability Statement

The raw data supporting the conclusions of this manuscript will be made available by the authors, without undue reservation, to any qualified researcher.

## Ethics Statement

This study was carried out in accordance with the recommendations of the University of Windsor Research Ethics Board with written informed consent from all participants. All participants gave written informed consent in accordance with the Declaration of Helsinki. The protocol was approved by the University of Windsor Research Ethics Board.

## Author Contributions

SH, RD, MG, JB, and PW contributed to the conception, design, and data collection for the study. All authors were involved in the data analysis process. Specifically, RD and MG led the analysis of the 2013 data, while SH and JD led the process for the 2017 data. SH and JD wrote the first draft of the manuscript. All authors contributed to manuscript revision and read and approved the submitted version.

### Conflict of Interest

The authors declare that the research was conducted in the absence of any commercial or financial relationships that could be construed as a potential conflict of interest.

## Footnotes

^1^We use the term “neoliberal” with caution because we know it is a contested term in the literature. In this study, we are using neoliberalism to refer to the current and dominant economic and political discourse (or “a sort of stand-in term for the political-economic zeitgeist”; Peck, 2010, p. 14) that pervades all aspects of society “to the point where it has become incorporated into the common-sense way many of us interpret, live in, and understand the world” (Harvey, 2005, p. 3).

^2^For the purpose of this study, we are using Gard et al.’s (2018) definition of policy as a “catch-all to talk about a range of phenomena such as official government programs and statements, educational curricula, existing and proposed public health interventions, the pronouncements of experts, and even media commentary” (p. 69).

^3^Gerber et al. (2018) offer the following example in target immediacy and local social comparison information: “…although comparison information about other students at a university may have effects on a person’s self-evaluation, those effects can easily be removed or muted by comparison information about the other students in one’s dormitory, and those effects can be removed or muted by comparison information about the students in one’s suite” (p. 179).
